# Competing risk multistate censored data modeling by propensity score matching method

**DOI:** 10.1038/s41598-024-54149-y

**Published:** 2024-02-22

**Authors:** Atanu Bhattacharjee, Gajendra K. Vishwakarma, Abhipsa Tripathy, Bhrigu Kumar Rajbongshi

**Affiliations:** 1 Division of Population Health and Genomics, Medical School, University of Dundee, Dundee, UK; 2 Department of Mathematics and Computing, Indian Institute of Technology, Dhanbad, India

**Keywords:** Multistate model, Competing risk, Non parametric estimation, Censoring, Propensity Score, Cancer, Medical research, Risk factors

## Abstract

The potential contribution of the paper is the use of the propensity score matching method for updating censored observations within the context of multi-state model featuring two competing risks.The competing risks are modelled using cause-specific Cox proportional hazard model.The simulation findings demonstrate that updating censored observations tends to lead to reduced bias and mean squared error for all estimated parameters in the risk of cause-specific Cox model.The results for a chemoradiotherapy real dataset are consistent with the simulation results.

## Introduction

Survival analysis refers to the statistical analysis of data that is measured from a particular time point until the occurrence of a specific event of interest or the attainment of a predetermined endpoint. It provides intuitive results concerning the period for events of interest, which is not confined to death but includes some other events. These events can be adverse, like the recurrence of the disease, or favorable, like recovery or discharge from the hospital. Therefore specialized techniques are devised to analyze such data with more efficacy. In cancer follow-up studies, multiple endpoints are observed, such as relapse of the disease, progression of the disease, and death status of the patient. Researchers frequently use the composite endpoint, which is defined as the time until either death or any of the nonfatal events whichever occurs first, and the information is incorporated in the final state^1^. In practical application, events typically arise in a sequential order, wherein the first occurrence of a disease is followed by its progression, culminating in eventual mortality. Survival time can refer to both the time survived from complete remission to recurrence and progression or overall time survived from diagnosis to death^2^.

**Figure 1:**
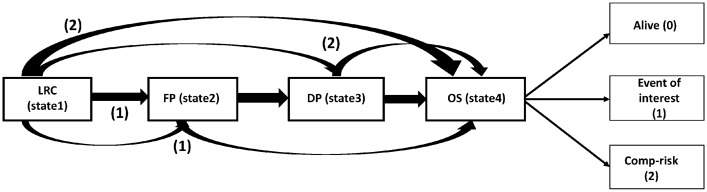
Multi-state survival model for right censored data in presence of competing risk.

In cancer studies, patients commonly go through multiple stages of the disease. In order to effectively analyse and understand the disease progression, researchers often employ the use of a multistate model (MSM) to analyse and interpret the associated data. An MSM is a continuous-time stochastic process that allows individuals to move among a finite number of states. A state can be transient if transitions emerge, and it is absorbing if no transition occurs. The primary transition pattern is modeled as a two-stage transition from “alive” to “death” state. Similarly, the illness-death model is another MSM that has been widely used in oncology studies to characterize disease development and also to investigate the rate of mortality^3^. However, the alive status can be further classified into two or more intermediate phases, each corresponding to different stages of illness^3^. The competing risk model is a particular case of MSM, which extends the basic mortality model of survival data by allowing each individual to die for numerous other reasons. Our method incorporates a multistate modeling approach, which distinguishes between individuals who have survived the nonfatal event and those who have died^4^. Our data contains four states, namely Loco Regional Control (LRC; state 1), First Progression (FP; state 2), Distant Progression (DP; state 3), and the final absorbing state (Death; state 4).LRC is defined as stopping the growth of cancer at its origin, hence being the state 1. Similarly, first and distant progression describes cancer progress in the local region and, thereby, in a remote region.LRC rates are often studied after providing treatments like chemotherapy to the patients. Similarly, the time duration until the final state is known as overall survival (OS). Conventionally, we consider the FP is followed by DP. Transitions are allowed from state 1 to state 2, 3, 4 progressively, however backward transition is restricted given in Fig. 1. In addition to the event-free survival probability, which has been the focus of composite endpoint analysis, the chance of being in each of these states can also be estimated^5,6^.

Often such data arise in oncology research when individuals are at risk of failure for K different reasons^7^. The study of such data necessitates a specific methodology that seeks to accurately assess the marginal probability of an event when there are several competing hazards. Conventional methods like the Kaplan–Meier survival function are the most commonly used survival estimate in time-to-event data in the absence of competing events^7,8^. However, the use of the Kaplan–Meier estimator in the presence of competing risk most often results in an upward bias of estimation of incidence function^9–11^. Therefore, we require specialized techniques to account for the competing events separately. The Cumulative Incidence Function (CIF), which predicts the marginal probability for each competing event, is the most popular alternative way for analyzing competing event data. CIF can be defined as the probability of failure from a certain cause before time t, in the presence of all other causes, and can be represented as a function of individual cause-specific hazard rates. To incorporate competing events in the Cox Ph model, there are two different types of hazard functions defined for the analysis of competing risk data, such as cause-specific hazard function and sub-distribution hazard function^12^. The cause-specific hazard model is a statistical method used to assess the impact of covariates on the rate of occurrence of specific events of subjects who have not experienced the event of interest at the current time. On the other hand, the sub-distribution hazard ratios derived from the Fine-Gray model describe of the relative impact of covariates on the sub-distribution hazard function^13^. Also, the stratified hazard function can be directly modeled through regression equations based on earlier work of Gray^14^ and Pepe^15^.

One challenging aspect of survival analysis is that, by the end of the study, only a few individuals experience the event, a phenomenon known as censoring. The exact survival time remains unknown for a specific group of individuals. Censoring is common in observational studies, necessitating the use of standard statistical methods to evaluate censored data, which relies on various assumptions about censoring^16,17^. However, if other factors influence censoring, accurate estimation of treatment effects becomes a concern, particularly when assuming that censoring is independent^18^. Any false assumption about censoring can lead to biased parameter estimation in the model. The survival function may be overestimated in the presence of a positive correlation between failure and censoring, or underestimated with a negative correlation^18^. In this study, we specifically address right-censoring cases and employ the Propensity Score Matching (PSM) technique to mitigate this issue.

Rosenbaum and Rubin proposed a methodology for assessing observational studies, wherein the likelihood of a subject belonging to a treatment (exposure) group is computed based on the covariates evaluated for that particular subject^19^. This score was termed as “propensity score”. The propensity score is commonly estimated through logistic regression, where the relationship between treatment (exposure) status and observable features (covariates) is examined by regression analysis. The propensity score (PS) can include a greater quantity of background covariates as it utilizes these covariates to estimate a numerical value^20^. After estimating the propensity score one of the methods of using this score to control the covariates is matching. In PSM a subject from the treatment group, sometimes referred to as the exposure group, is chosen randomly and subsequently paired with an untreated subject based on their propensity score^19^. One-to-one propensity score matching is the most prevalent method when treated and untreated subjects have similar scores^21^. Matching can be conducted either with or without replacement. However, matching with replacement has the potential to reduce bias and is particularly advantageous in situations where the availability of control subjects is limited^22^. PSM is a popular analytic method for removing the effects of confounding due to measured baseline covariates when using observational data to estimate the effects of treatment^23^. The propensity score method has many desirable properties like balancing the confounders between treatment and control groups to obtain conditional independence between them^24^. This method is used in different ways like score matching in case–control studies and Inverse Probability of Treatment Weighting (IPTW) to measure of effect frequently reported in randomized control trials^25^. Similarly, for observational research, Rosenbaum and Rubin proposed a propensity score method to quantify the causal impact of the treatment^19^. In the case of right-censored response data PSM method has been used for robust estimation of treatment effects^26^. In this article, we propose the use of PSM in updating individual patients’ information about censoring for different types of competing events and thereby analyzing and comparing the competing risks in the data set. The proposed method is elaborated in the manuscript.

## Methods

### Multistate model

The MSM serves as an effective method to depict complex clinical processes over time. It is a versatile framework commonly employed in clinical conditions characterized by increasing disease severity and imminent mortality. MSMs are typically assumed to follow the Markov process, aiding in the explanation of disease progression and all potential transitions. This approach also identifies previously visited states, enhancing visual representation^27^. While MSMs can accommodate various endpoints, our study specifically considers death as an absorbing state.

It is a continuous-time stochastic process, represented by $X_{t}$, $t\in T$, where an individual is allowed to transit from an initial state at $T=0$ to several intermediate states and finally to the absorbing state at $T=t$. Let *X*(*t*), be a finite state with state space $S=\{1,2,\ldots ,q\}$. The process has a initial distribution defined as, $\pi _{i}(0)= Prob(X(0)=i, i \in S$. The transition probability from state i to j is considered to be following the Markovian process and is defined as;

1 $$\begin{aligned} P_{ij}(s,t)=P(X(t)=j|X(s)=i)\;;i,j\in S,s,t\in T) \end{aligned}$$

Covariates can be incorporated into the model through transition intensities in a Cox proportional hazard (Cox PH) model to explain the effect of covariates in different transitions. The Cox Ph hazard equation for the transition from *i* to *j* can be written as,

2 $$\begin{aligned} h_{ij}(t|\textbf{z})=h_{ij0}(t)exp(\beta _{ij}\textbf{z}') \end{aligned}$$

where $T=t$, represents the time when the individual reaches state j, from state i. $\textbf{z}$ is the vector of covariates, and $h_{ij0}$ is the baseline hazard of transition $i->j$. This is also known as the transition-specific Cox PH model. Intermediate events frequently alter the path of disease progression, which results in a shift in the role of some prognostic factors following that event.

Survival data mostly contains censored information, and in this study, we’ll be focusing on right censored data. For *n* no. of individuals let $t_{l}$ be the event time and $c_{l}$ be the censoring time. The primary assumption for right censored data is censoring distribution and event time distribution are independent of each other. Let $\delta _{l}$ be the event indicator. Then $\delta _{l}=1$ if $t_{l} \le c_{l}$, and 0 otherwise.

### Multistate with competing risk

The presence of competing events in survival data prevents the occurrence of the primary event of interest^12^. A MSM in the presence of competing risk has an initial state, q no. of intermediate events, and one final state which is further split into three possible events such as censored, death due to the primary event, and the competing event. Individuals may either progress to the endpoint through several intermediate stages or directly move to the absorbing state. In such cases, if an individual dies with no intermediate events in his prognosis, then such a death is termed a competing risk or competing event; otherwise, it is known as a primary event. These two events are mutually exclusive, as an individual who directly progresses to the death stage cannot experience intermediate states and vice versa.

Let the type of events at the final state be denoted by $k= 0,1,2$, where death due to local progression is denoted by $k=1$ and competing event denoted by $k=2$. Cause-specific hazard rate describes the instantaneous rate of occurrence of the kth event in subjects who are currently event-free and have not yet experienced any type of event.

It is defined as,

3 $$\begin{aligned} \lambda _{k}(t) = \lim _{\Delta t\rightarrow 0} \frac{P(t \le T < t+\Delta t, D = k | T \ge t)}{\Delta t} \end{aligned}$$

where D represents the type of event; $k=1,2$.

Similarly, the cumulative cause-specific hazard for individual causes is given by,

4 $$\begin{aligned} A_{k}(t) = \int _{0}^{t} \lambda _{k}(u)du \end{aligned}$$

for $k=1,2$. The probability of occurrence of each type of event k up to a given time t is called as ’Cumulative Incidence Function’ (CIF). CIF is also defined as $1-S(t)$, which calculates the probability of an event occurring while considering competing risk events. The CIF of kth cause is given by,

5 $$\begin{aligned} F_{k}(t) = P(T \le t, D = k) = \int _{0}^{t}\lambda _{k}(u)P(T\ge u)du = \int _{0}^{t}\lambda _{k}(u)[-A_{k}(u)]du \end{aligned}$$

for $k=1,2$. CIF is not being calculated for $k=0$ as it represents censored individuals. However, as there is no one-to-one correlation between hazard and cumulative incidence, the influence on hazard cannot be immediately related to the effect on CIF^28^. Therefore Fine-Gray sub-distribution hazard model was developed to link covariates to cumulative incidence directly. The sub-distribution hazard model developed by Fine-Gray^29^. It describes the instantaneous risk of failure from *kth* event in subjects who are currently event-free as well as those who have previously experienced a competing event^14^. The **cuminc** function package **cmprsk** can estimate the CIFs from different causes of failure and allow comparison between groups.

However the competing events can be incorporated in Cox PH model as well and defined as,

6 $$\begin{aligned} h_{k}(t|z) = h_{k0}(t) exp(\beta _{k}z) \end{aligned}$$

where $h_{k0}(t)$ is the baseline of the cause-specific hazard function and the vector $\beta _{k}$ represents the covariate effect on the effect of interest.

### Propensity score matching method

Information is often incomplete due to censorship, and there are competing risk events. This means some people may have died, but we don’t have their exact status. This information gap can affect comparing treatment effects. PSM is a method to create a comparable control group by matching treated and untreated individuals based on similar characteristics. We use the matching technique in PSM for individuals with incomplete survival information. For censored individuals, PSM matches them with actual events using propensity scores based on their characteristics. Matching minimizes the impact of measured variables on the comparison of treatment effects^30^. Our approach involves updating individual patient information about censoring for different events and applying various models for multi-state analysis to estimate hazard rates with covariates. In our model, we have individuals with certain characteristics and consider four states: LRC, FP, DP, and Death. We use the Bayesian Cox Proportional Hazards model to obtain regression parameters for covariates. Propensity scores are calculated based on the sign of the regression parameters. We define ‘closeness’ in terms of propensity scores using a distance metric, such as the Euclidean distance. This score measures the similarity between a censored individual and actual dead individual. Another way used to explain this is Rosenbaum and Rubin’s proposed employing a caliper equal to 0.25 times the propensity score. This approach has been demonstrated to effectively mitigate 98 $\%$ of the bias resulting from measured covariates^31^. After calculating the propensity score, we update censored individuals based on a threshold probability. If the propensity score is high, the censored person is less likely to die in real life. This updated information helps analyze the data effectively. Censored individuals are updated to competing risk states using transition probabilities. For example, an individual may transition from being censored to competing risk 1 or 2. In summary, our proposed PSM method uses propensity scores to match individuals, update censored information, and analyze survival data with competing risks and covariates.

Let there be n individuals with m covariates denoted by $X_{1},X_{2},\ldots ,X_{m}$. The number of individuals at $i\text{ th }$ state as $S_i;\;i=\{1,2,3,4\}$. In our model, we have considered the four states to be LRC, FP, DP, and Death. Regression parameters of the m covariates $\varvec{\beta }'=(\beta _0,\beta _1,\ldots ,\beta _m)$ are obtained through the Bayesian Cox Ph model using the Markov chain Monte Carlo (MCMC) method. Depending on the sign of the $\beta$ value propensity score is calculated as given below; $\textbf{X}_{min}^{T}=(x_{1min},x_{2min},\ldots ,x_{mmin})$ where

7 $$\begin{aligned} x_{lmin} = {\left\{ \begin{array}{ll} \min \{x_{11},x_{12},\ldots ,x_{1n}\} \;;\;\text {if}\;\; \beta _l>0\\ \max \{x_{11},x_{12},\ldots ,x_{1n}\} \;;\text {if}\;\; \beta _l\le 0\\ \end{array}\right. } \end{aligned}$$

where $l=1,2,\ldots ,m$;$\varvec{\beta }'=(\beta _0,\beta _1,\ldots ,\beta _m)$ are the regression parameters obtained from Bayesian Cox PH model using Markov Chain Monte Carlo (MCMC). Using the Bayesian Cox Ph model on censored individuals at state $i=1,2,3,4$, we can identify the covariates that are impacting the deaths. The point $X_{min}$ is set up in such a way that all the covariates that are contributing significantly to the hazard are placed to the right of the threshold value, and all other covariates are placed to the left.

The distance function or metrics specify the distance between points in space. The similarity between two points $\textbf{X}_{min}$ and $\textbf{X}_k$ is obtained using the different distance metric which is referred as propensity score, where $\textbf{X}_r$ is the covariate information of the *rth* individual who is censored. It is defined as the measure of similarity between a censored individual which is considered to be alive and actual dead individuals. The propensity score for $r\text{ th }$ censored individual is defined as

8 $$\begin{aligned} \Delta _{r}=d(\textbf{X}_{r},\textbf{X}_{min}) \end{aligned}$$

where $d(\cdot )$ is distance metric for the *m* dimensional space. The metric $d:\mathbb {R}^{m}\times \mathbb {R}^{m}\rightarrow \mathbb {R}$ satisfies the following condition

1. $$d(\textbf{X},\textbf{Y})=0$$ iff $$\textbf{X}=\textbf{Y}$$
2. $$d(\textbf{X},\textbf{Y})=d(\textbf{Y},\textbf{X})$$
3. $$d(\textbf{X},\textbf{Y})\le d(\textbf{X},\textbf{Z})+d(\textbf{Z},\textbf{Y})$$ where $$\textbf{X},\textbf{Y},\textbf{Z}\in \mathbb {R}^{m}$$

Euclidean distance metric has been used in this study to compute the propensity score.Using this method the distance between two m-dimensional vector $\textbf{X},\textbf{Y}$ is given by $d(\textbf{X},\textbf{Y})=\sum _{v=1}^{m}w_v(x_{v}-y_{v})^2$.If we consider $w_v=1$ then it is unstandardized and standardized by S.D when $w_v=\frac{1}{s_v^2}$. The propensity score for *rth* individual who is censored using Euclidean distance metric is given by

9 $$\begin{aligned} \Delta _{r}^{e}=d(\textbf{X}_{r},\textbf{X}_{min})=\sum _{v=1}^{m}w_v(x_{vmin}-x_{vr})^2 \end{aligned}$$

where $\textbf{X}_{min}^{T}=(x_{1min},x_{2min},\ldots ,x_{mmin})$ and covariate information for *rth* individual who is censored is $\textbf{X}_{r}^{T}=(x_{1k},x_{2k},\ldots ,x_{mk})$.

Though censored individuals are considered to be alive, the caliper method uses the propensity score to match censored alive with real death. A threshold probability has been assumed to compare the propensity scores of censored individuals with that of actual death cases. If the propensity score is high, it is less likely that a censored person will die in real life. For a given value of threshold probability *p*, the death status for the censored individual gets updated as follows,

$$\begin{aligned} I_r= {\left\{ \begin{array}{ll} 1 \;;\;\text {if}\;\; p>1-F(\Delta _{r}<\delta )\\ 0 \; ;\;\; \text {otherwise} \end{array}\right. } \end{aligned}$$

where $\delta$ is the $p\text{ th }$ quantile of $\Delta _r$ and $\Delta _r$ is the corresponding Euclidean distance metric. More is the p value from the cumulative probability, and higher is the chance that the individual is updated to status 1. After updating the censored information of the individuals and bifurcating them into dead or alive, we can apply the required functions and analyze the data accordingly.

Individuals get updated from censored cases (0) to competing risk 1, as well as competing risk 2. Let the transition be denoted by $\tau _{0k}$, where $k=1,2$ represents the competing events.

### Estimation

Assuming the distribution of censored times to be non-informative that is parameters of event times distribution and the censored time distribution are two different sets, estimated values of the parameters are obtained by maximizing the likelihood function obtained from the risk set at the events times. The effect of covariates for transition $i-j$, can be modeled through the Cox PH model given by,

10 $$\begin{aligned} h_{ij}(t|\textbf{z})=h_{ij0}(t)exp(\beta _{ij}\textbf{z}') \end{aligned}$$

Alternatively, we can write the model as,

11 $$\begin{aligned} h_{ij}(t)=h_{ij0}(t)exp(\beta _{ij}'\mathbf {z_{ij}}) \end{aligned}$$

where $z_{ij}$ is the vector of covariates specific to the transition $i->j$, specifically designed for individual based on his covariates. Let *G* be the set of all possible transitions $i->j$. The estimated value of the parameters can be obtained by maximizing the partial likelihood function as given below,

12 $$\begin{aligned} L(\varvec{\beta })=\prod _{ij\in G}L_{ij}(\varvec{\beta }_{ij}) \end{aligned}$$

Here in the presence of competing risk, for each independent cause k, the regression coefficient $\beta _{k}$ can be estimated by maximizing the modified partial likelihood. The likelihood function is written as,

13 $$\begin{aligned} L(\beta _{k}|\textbf{z}_1,\textbf{z}_2,\ldots ,\textbf{z}_m) = \prod _{l=1}^{n} (h_{k0}(t_l)e^{\varvec{\beta }_k\mathbf {z_l}})^{\delta _l}e^{-H_{k0}(t_l)e^{\varvec{\beta }_k\mathbf {z_l}}} \end{aligned}$$

where n denotes the total no. of individuals. Let $Z_{ij,k}$ denote this vector for competing event k, then the estimates of $\beta$ vector can be obtained by maximising the generalized the Cox partial likelihood model given by,

14 $$\begin{aligned} L(\beta _{k}|\textbf{z}_1,\textbf{z}_2,\ldots ,\textbf{z}_m) = \prod _{i->j} \prod _{d_{ij,k}=1}^{2} \frac{exp(\beta ' z_{ij,k})}{\sum _{l \in R_{i}(t_{ij},k)}exp(\beta ' Z_{ij,l})} \end{aligned}$$

where $t_{ij},k$ is either failure or censoring time for competing event *k*, for transition $i->j$. $d_{ij,k} = 1$ if an individual has an event for transition $i->j$ for competing event *k*, 0 otherwise. $R_{i}(t)$ is the risk set for the set of individuals in state *i* at time t. The estimate of the cumulative baseline hazard using the Nelson–Aalen estimator is given by,

15 $$\begin{aligned} H_{ij,0} (t) = \sum _{t_{ij,k}\le t} \frac{d_{ij,k}}{\sum _{l \in R_{i}(t_{ij},k)}exp (\beta ' Z_{ij,l})} \end{aligned}$$

For calculation purposes plugging in the non-parametric estimator for baseline and cumulative baseline hazard $h_{k0}(t)=\frac{\delta }{\sum _{l \in R_{i}(t_{ij},k)}e^{\varvec{\beta }_k \mathbf {z_j'}}}$ in Eq. (13), we get the estimated parameters by maximizing the partial likelihood function given below

16 $$\begin{aligned} L(\beta _{k}|\textbf{z}_1,\textbf{z}_2,\ldots ,\textbf{z}_m) = \prod _{l=1}^{n}\left( \frac{e^{\varvec{\beta }_k\textbf{z}_l}}{\sum _{l \in R_{i}(t_{ij},k)}e^{\varvec{\beta }_k}\textbf{z}_j'}\right) ^{\delta _l} \end{aligned}$$

where *R*(*t*) represents the risk set which consists of individuals at risk at event time t, $\delta _l$ be the censoring status of *lth* individual. Throughout we have assumed that $z_{i}$ does not include any intercept, as it is not estimable in Cox partial likelihood. Also $z_{i}$, survival time $T_{i}$, censoring time $C_{i}$ are independent of each other. For completely observed data maximum partial likelihood estimate (MPLE) is defined as

17 $$\begin{aligned} \hat{\beta } = arg max_{\beta }L(\beta _{k}|\textbf{z}_1,\textbf{z}_2,\ldots ,\textbf{z}_m) \end{aligned}$$

According to Cox, the conventional features of maximum likelihood estimation for large samples can be extended to the partial likelihood^32^. The derivatives of the likelihood concerning $\beta$, both the first and second derivatives, are consistent with those presented in Cox’s work from 1972, except the risk set definition and the arguments of the independent variables^33^. Partial likelihood estimates of the coefficients $\beta _k$ can be obtained using numerical methods of the Newton–Raphson type. MPLE can be computed using standard statistical software nowadays.

## Simulations

### Data simulation

Simulation studies are frequently used to assess how the existing and currently developed statistical models perform in the analysis of data^34^. Therefore it is necessary to generate a new dataset that closely mimics a real-life event. The exponential distribution, which assumes a constant underlying hazard function, or the Weibull distribution, which assumes a monotonically growing or decreasing hazard, are utilized and implemented with the methodologies in the survival analysis^35^. Even though the Cox model is the most widely used method of survival analysis, a semi-parametric model cannot be used to simulate a dataset^36^. Therefore, a common approach has to be made to simplify the parametric assumptions about the distribution of event times. A general method known as the cumulative hazard inversion method allows one to simulate event times using any parametric formulation for the baseline hazard in a proportional hazards data generating model^35,37^. To overcome the difficulties in situations where cumulative baseline hazard is not invertible, Crowther and Lambert proposed an algorithm that nested numerical integration inside numerical root finding^38^. The **“simsurv”** package allows one to simulate event times from standard parametric distributions (exponential, Weibull, and Gompertz), two-component mixture distributions, or a user-defined hazard, log hazard, cumulative hazard, or log cumulative hazard function. Simulated datasets are used to compare the effects of various prognostic factors and calculate the uncertainty of model predictions, such as transition probabilities in multistate models and the impact of competing risks. We used **“mstate”** packages available in CRAN R to simulate survival data with multiple states in our study. Each simulated dataset contains 200 individuals in our research. We considered two different competing risk events and generated two other survival times corresponding to both the risks, namely $s_{1}$ and $s_{2}$. Survival times were generated using the **“simsurv”** package listed in CRAN R.

Cox Ph model is widely used to analyze the impact of covariates on survival time. Therefore, to simulate survival time Cox Ph is used as given below

18 $$\begin{aligned} h(t)=h_0(t)exp(x_1\beta _1+x_2\beta _2+x_3\beta _3) \end{aligned}$$

where $h_0(t)$ is the baseline hazard. We have considered that survival time *t* follows Weibull distribution, now the baseline hazard takes the form $h_0(t)=\gamma \lambda t^{\lambda -1}$. Further, we assume *S*(*t*) to follow uniform distribution, $~ U\sim U(0,1)$, in order to obtain the simulated survival time as,

19 $$\begin{aligned} t&=S^{-1}(u)=H^{-1}(-\log u) \end{aligned}$$

Now using the cumulative hazard function $H(t)=\lambda t^{\gamma }$ in the above equation we get the simulated survival time from the Weibull distribution with parameters $\lambda ,\gamma$ as given by

20 $$\begin{aligned} t=\left[ \frac{-\log u}{\lambda e^{\textbf{x}\varvec{\beta }}}\right] ^{1/\gamma } \end{aligned}$$

The parameter values for the Cox regression for survival time $s_{1}$ were chosen arbitrarily as follows $\beta _1= -0.5, \beta _2=0.01, \beta _3=0.02, \gamma =1.5,\lambda =0.1$. Similarly, for survival time $s_{2}$ the parameters values are chosen to be $\beta _1= -0.5, \beta _2=0.43, \beta _3=0.03, \gamma =1.5,\lambda =0.1$. $X_{1},X_{2},X_{3}$ are the covariates can be both continuous and categorical. Yet, in our simulation study, we have considered $X_{i}$ to be continuous at the baseline level. A baseline covariate in the context of survival data is defined as a qualitative or quantitative variable that is measured or observed before randomization and is expected to influence the primary outcome variable to be examined. When investigating the relationship between a survival outcome and covariates, statisticians frequently consider the covariate’s baseline value, which fails to take into account the relationship between survival outcome and changes in the values of the covariates. In this model, we have generated a continuous set of baseline covariates that follows the normal distribution.

Censoring time was taken to be 5 years, say. Then comparing the two survival times with censoring time, we generated the status of the individuals as 0, 1, and 2. A total of 100 such datasets are simulated. Using the Cox PH model, we calculated the $\beta$ coefficients for each covariate and therefore calculated bias, bias, and mean score error (MSE), respectively.

### Analysis of simulated data

Our method includes a competing risk modeling approach, which discriminates between individuals who died due to cancer and those who have died due to causes other than cancer. Our data contains two competing risk events, namely death due to cancer denoted by status = 1 and death caused due by reasons other than cancer denoted by status = 2. Two separate survival times are generated for both the causes denoted by $s_{1}$ and $s_{2}$ respectively. Censoring time is assumed to be five units, i.e., the study ends after 5 years/months. Survival time $s_{1}$ is compared with censoring time and accordingly status for $s_{1}$ is generated as 0 and 1 denoted as $st_{1}$. Similarly, survival time $s_{2}$ is compared with censoring time, and accordingly status for $s_{2}$ is generated as 0 and 2 denoted as $st_{2}$. Then comparing $st_{1}$ and $st_{2}$ final status is generated as 0, 1 and 2. Applying Cox PH function separately for $st_{1}$ and $st_{2}$, we can find out the hazards for each cause separately.

**Table 1:** Simulation results for 100 dataset consist of 200 subjects each with true parameter value $\beta _1=-0.5,\beta _2=0.01,\beta _3=0.02$ and $\beta _1=-0.5,\beta _2=0.43,\beta _3=0.03$.

Parameters	Mean	Bias	MSE	Mean	Bias	MSE
	Without propensity score method ($\tau _{01}$)			Without propensity score method ($\tau _{02}$)		
$\beta _1(x_1)$	− 0.522	− 0.022	0.209	− 0.519	− 0.019	0.059
$\beta _2(x_2)$	0.014	0.004	0.002	0.015	0.025	0.003
$\beta _3(x_3)$	0.017	0.002	0.002	0.014	− 0.185	0.002
	With propensity score method			With propensity score method		
$\beta _1(x_1)$	− 0.504	− 0.004	0.156	− 0.510	− 0.010	0.003
$\beta _2(x_2)$	0.048	0.005	0.039	0.048	0.0054	0.018
$\beta _3(x_3)$	0.031	0.001	0.002	0.025	0.005	0.001

In an MSM with more than one intermediate event, censoring can take place in any of the states; as a result, to update such cases, we take the help of the PSM method. We assumed an arbitrary value of 0.9 for the threshold probability. If the cumulative distribution function of the propensity score value is greater than or equal to 0.9, the status of a censored individual is updated as dead. PSM method is applied to update those individuals, and further split them into dead and alive. A cause-specific Cox Ph model was used to select the effect of different prognostic factors on the propensity score. The R package **“survival”** was used for the analyses of the cause-specific Cox PH model. Each simulated data set was analyzed before and after applying the propensity score matching separately for both competing events. Results of the simulated data are shown in Table 1. Regarding competing event 1, the mean estimated regression coefficients for covariate $x_2$ obtained before and after updation of the dataset using propensity score matching are 0.0144 and 0.0486, respectively. The MSE of the regression coefficient $\beta _1,\beta _3$ reduced while using propensity score matching. Similarly, the bias for estimator $\beta _3$ reduces to 0.0017 from 0.0023 after applying the PSM technique. Likewise, for competing event 2, the bias for regression coefficient $\beta _2$ reduced from 0.0255 to 0.0054, MSE of $\beta _1$ reduced to 0.0031 after the application of PSM. Using PSM we have proposed an approach that reduces the bias and MSE for estimating measures of effect in the Cox PH model in competing risk settings. Thereby we can say PSM method gives better estimates of regression coefficients.

## Analysis of chemoradiotherapy dataset

The proposed method is applied to the Chemoradiotherapy dataset^39^. This data frame contains information of 536 individuals followed up from 2012 to 2018. The listed variables are id, four different states, and a few covariates. The four different states are namely Loco Regional Control (LRC) as state 1, First Progression (FP) as state 2, Distant Progression (DP) as state 3, and Overall Survival (OS) as state 4 as shown in Fig. 1. The date of each event is given in the dataset, comparing the date of events with the last follow-up date status for each state is generated, and a separate column for status is generated.

It consists of the information on causes of death due to cancer or reasons other than cancer. The covariates included in the study are baseline hemoglobin level, baseline sugar level, baseline serum creatinine, baseline serum albumin, baseline serum sodium, and baseline serum potassium denoted as cov1, cov2, cov3, cov4, cov5, and cov6, respectively. The data also provides information about the gender of each patient, which is further used in computing hazard rates for each gender separately. Individuals who progress directly from LRC to death or LRC to death without FP taking place are termed as having competing risk 2, whereas those individuals who proceed to death after a local progression taking place are classified as competing risk 1 (Fig. 1).

**Table 2:** Results of cause specific Cox regression applied on Chemoradiotherapy data.

Parameters	$\beta$	$exp(\beta )$	se$(\beta )$	z	p	$\beta$	$exp(\beta )$	$se(\beta )$	z	p
$\tau _{01}$	Without propensity score method					With propensity score method				
cov1	− 0.061	0.940	0.055	− 1.100	0.271	− 0.067	0.934	0.055	− 1.220	0.222
cov2	0.001	1.001	0.003	0.463	0.643	0.001	1.001	0.003	0.434	0.664
cov3	− 0.006	0.993	0.004	− 1.623	0.104	− 0.006	0.993	0.004	− 1.570	0.116
cov4	− 0.545	0.579	0.196	− 2.778	0.005	− 0.525	0.591	0.196	− 2.671	0.007
cov5	− 0.005	0.994	0.033	− 0.155	0.877	− 0.007	0.992	0.033	− 0.230	0.818
cov6	− 0.088	0.915	0.201	− 0.401	0.659	− 0.093	0.910	0.200	− 0.468	0.639
Male	0.386	1.472	0.270	1.420	0.152	0.351	1.420	0.264	1.327	0.184
$\tau _{02}$	Without propensity score method					With propensity score method				
cov1	− 0.115	0.890	0.076	− 1.507	0.131	− 0.081	0.921	0.065	− 1.248	0.219
cov2	− 0.000	0.999	0.005	− 0.157	0.875	− 0.002	0.997	0.004	− 0.476	0.633
cov3	− 0.011	0.988	0.006	− 1.809	0.070	− 0.006	0.993	0.005	− 1.139	0.254
cov4	− 0.731	0.481	0.252	− 2.89	0.003	− 0.597	0.550	0.235	− 2.541	0.011
cov5	− 0.101	0.903	0.042	− 2.40	0.016	− 0.104	0.901	0.034	− 3.041	0.002
cov6	− 0.291	0.747	0.285	− 1.01	0.308	− 0.283	0.752	0.246	− 1.152	0.249
Male	0.441	1.554	0.383	1.152	0.249	0.096	1.100	0.298	0.322	0.747

**Figure 2:**
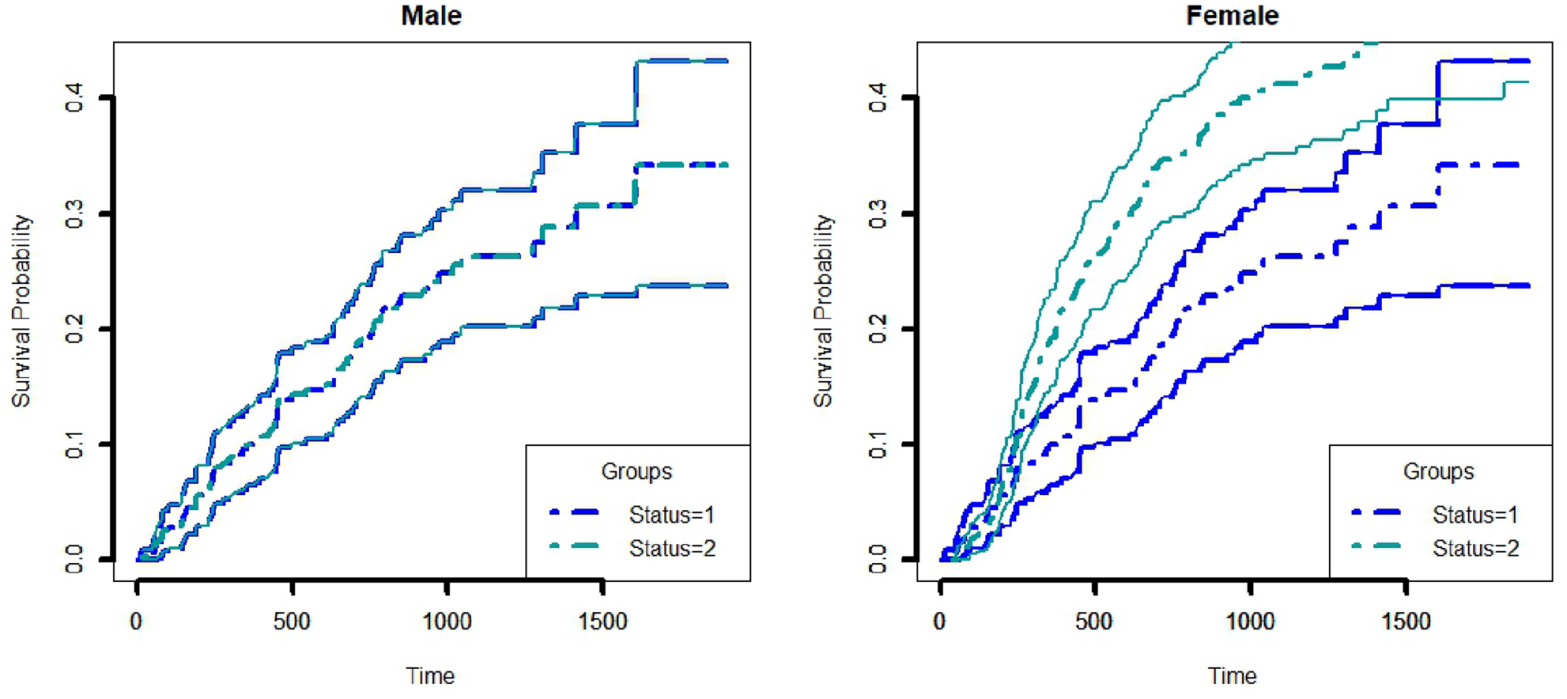
Cumulative incidence curve for male and female using nimodata. 1, 2 represents competing risks.

**Figure 3:**
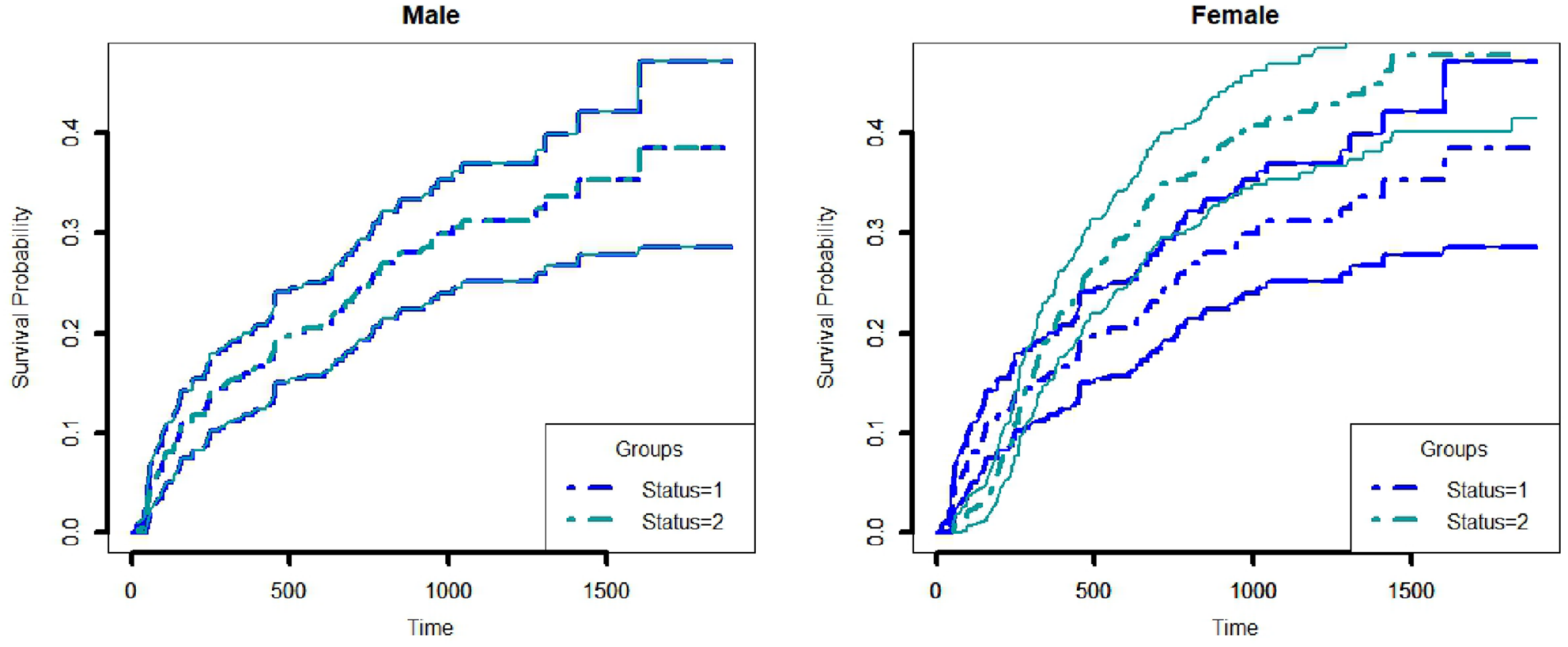
Cumulative incidence curve for male and female using updated nimodata by propensity score matching. 1, 2 represents competing risks.

Cause-specific Cox proportional model is used to analyze the data. The death status of the censored cases at the final state is updated using a propensity score matching with a threshold probability of 0.9. Results of the cause-specific Cox proportional hazard model of the dataset before and after applying PSM are shown in Table 2. In the dataset, there were 154 individuals of competing risk 1, which gets updated to 156 after applying the PSM method. Similarly, for competing risk 2, 75 individuals were updated to 103 individuals. Both males and females with competing risk 1 have a higher cumulative incidence rate compared to those with competing risk 2 in the original dataset (Fig. 2). With the application of PSM, after updating the censored information difference between the CIF curves can be seen from Fig. 3, especially for female individuals, the cumulative incidence for competing risk 2 surpasses competing risk 1, for a certain period.

## Discussion

In cancer research, a variety of time-to-event endpoints can be studied, commonly known as composite endpoints^40^. Analysis of a single component among the competing events of time-to-event data yields more precise results^41–43^. The overall survival can also be considered as composite as it can be distinguished between cancer-related mortality and non-cancer mortality^44,45^. The intermediate events like, e.g., death after recurrence, progression, or metastasis can also be studied in a multistate framework^46^. For instance, the incidence of competing risk can be counted if a transition is from the initial state to the final competing event, without any progression^47^. Solely analysis of “progression” usually fails to account for competing risk events, which compromises the outcome^48^.

Survival analysis in the presence of competing events urges additional challenges for the investigator as the hazard function lacks one-to-one correspondence with the CIF^28^. Kaplan–Meier survival curve is not preferable to be used to estimate CIF for data with competing risk events, as it may overestimate the probability of an event. Hence Aalen–Johansen estimator is recommended. This article uses the cause-specific Cox PH model, a semi-parametric approach, to evaluate the effect of several covariates on survival after updating the censored information of individuals simultaneously. There are many such research articles on competing risk regression modeling that has been published in Clinical Cancer Research of late^49,50^. The competing risk modeling comprises of comparison of two groups of individuals who will develop the event over time with those who will not develop the event is mostly seen in various literature for time-dependent events^51^. However, the above statement violates the principle established by Andersen and Keiding, i.e., “Do not condition on future”^52^, in the analysis of survival data because it may give space to biasedness^53^. Within-subject cause-specific dependency for univariate and bivariate competing risk data is hardly identifiable non-parametrically, established by Tsiatis^54^ and^55^. The independence assumption within the subject is hardly testable for which the validity of parametric analysis is ambiguous^56^. Recently, much literature has focused on cause-specific hazard function (CSH) and cumulative incidence function (CIF) to avoid practical and theoretical difficulties^57^. Prentice et al., in a paper, established that CSHs and CIFs are estimable for competing risk data^58^.

The etiology of the competing event is investigated using the cause-specific hazard function to analyze the direct association of treatment with the instantaneous hazard of the event^47^. Many kinds of literature on competing risk also mentioned risk-specific latent times and their association with a single cause-specific hazard, eliminating the other competing risk^59^. This assumption calls for the in-dependency concept of competing risk events, which is beyond the scope of the article.

When we focus our interest on the course of the disease with composite endpoints, MSM becomes very useful to explain the structure. However, the study plan is always exigent. The available approaches are listed in a few literature^60^. A practical application of clinical trials with diabetes patients is documented in the literature by Schulgen for sample size selection^61^. The appropriate size of the sample plays a vital role in the analysis of clinical data. However, such data like MSM mostly suffers from censoring of individuals. There is a sample number of individuals whose death status is censored in the dataset. However, in reality, those individuals might die due to both cancerous and non-cancerous reasons. To update the censored information, this article uses the PSM technique to update the dataset and bifurcate them into both competing events. By doing so, we have also updated the sample size in both wings of competing risk events, thereby making the analysis more meaningful. A simulation study has been carried out with simulated data for a better understanding of the impact of competing risks on the survival and efficacy of the PSM method. Similarly, the application of simulation techniques to empirical data can be seen for complex endpoint structures^62^. Extending the methodologies of the current research to accommodate the semi-competing risks setup would be interesting. Censored information updating using propensity score is a topic that will be investigated further in the future.

## Conclusion

In the presence of competing events in the dataset, what method to be used for analysis is determined by the type of research question. Both cause-specific hazard models and sub-distribution hazard models can be used for competing risk analysis, with model-specific advantages. This article proposes the method of propensity score matching to match the score of censored individuals with those of non-censored cases and update their status to alive or dead. Thereafter cause-specific Cox PH models have been used for competing for risk events in MSM. Also, the competing risk events are analyzed using CIF separately for the risk factors. The simulation study shows that the mean square error and bias of the regression coefficient reduced after applying PSM. The same methodology is applied to the Nimotuzumab Chemoradiotherapy dataset, and a similar result is obtained. There were 154 individuals with competing risk 1 in the dataset. When the PSM approach was used, the number increased to 156. In the same way, 75 individuals were updated to 103 for competing risk 2.

## Data Availability

The data used here is not available publicly, however data can be obtained from the corresponding author on reasonable request.
